# Supplementary material for: Ultralow thermal conductivity from transverse acoustic phonon suppression in distorted crystalline α-MgAgSb
Source: Nat Commun. 2020 Feb 18;11:942. doi: 10.1038/s41467-020-14772-5 (PMC7029039; doi:10.1038/s41467-020-14772-5)
Supplement: Supplementary file 1 — Supplementary Information [file 41467_2020_14772_MOESM1_ESM.pdf]

SUPPLEMENTARY INFORMATION

Ultralow Thermal Conductivity from Transverse Phonon Suppression in Distorted Crystalline α-MgAgSb

Xiyang Li[1,2,3,4]*, Peng-Fei Liu[5]*, Enyue Zhao[1,4], Zhigang Zhang[2], Tatiana Guidi[6], Manh Duc Le[6], Maxim Avdeev[7], Kazutaka Ikeda[8], Toshiya Otomo[8], Maiko Kofu[9], Kenji Nakajima[9], Jie Chen[5], Lunhua He[1,5], Yang Ren[10], Xun-Li Wang[3], Bao-Tian Wang[5], Zhifeng Ren[11], Huaizhou Zhao[1] and Fangwei Wang[1,2,4]

[1]*Beijing National Laboratory for Condensed Matter Physics, Institute of Physics, Chinese Academy of Sciences, Beijing 100190, China.*

[2]*Songshan Lake Materials Laboratory, Dongguan 523808, China.*

[3]*Department of Physics, City University of Hong Kong, 83 Tat Chee Avenue, Hong Kong, China.*

[4]*School of Physical Sciences, University of Chinese Academy of Sciences, Beijing 101408, China.*

[5]*Spallation Neutron Source Science Center, Dongguan 523803, China.*

[6]*ISIS facility, Rutherford Appleton Laboratory, Chilton, Didcot, OX11 0QX Oxfordshire, UK.*

[7]*Australian Nuclear Science and Technology Organisation, Lucas Heights, NSW 2234, Australia.*

[8]*Institute of Materials Structure Science, High Energy Accelerator Research Organization (KEK), Tsukuba, Ibaraki 305-0801, Japan.*

[9]*Japan Proton Accelerator Research Complex, Japan Atomic Energy Agency, Tokai, Ibaraki 319-1195, Japan.*

[10]*X-ray Science Division, Argonne National Laboratory, Argonne, IL 60439, USA.*

[11]*Department of Physics and TcSUH, University of Houston, Houston, Texas 77204, USA.*

(Dated: 1/17/2020)

* These authors contributed equally.

Correspondence and requests for materials should be addressed to B. T. W. (email: wangbt@ihep.ac.cn) or to Z. F. R. (email: zren@uh.edu) or to H. Z. Z. (email: hzhao@iphy.ac.cn) or to F. W. W. (email: fwwang@iphy.ac.cn)

Supplementary Note 1: Rietveld refinement results of neutron diffraction data.

The temperature-dependent neutron diffraction data were measured at NOVA@J-PARC from 20 to 500 K for $MgAg_{0.97}Sb_{0.99}$ and $MgAg_{0.965}Ni_{0.005}Sb_{0.99}$, respectively, and the Rietveld refinement analysis was done using the Z-Rietveld program. Here, the atomic displacement parameters were refined under the constraints of all Ag atoms have the same thermal displacements and all atom occupancies are fixed. The refinement results are shown in Supplementary Fig. 1, Supplementary Table 1, and Supplementary Table 2. The 150 and 250 K data shown in Supplementary Fig. 1a, Supplementary Fig. 1c, Supplementary Fig. 1d, and Supplementary Fig. 1e are measured at GDDP@CSNS[1] for $MgAg_{0.97}Sb_{0.99}$ and $MgAg_{0.965}Ni_{0.005}Sb_{0.99}$, respectively. The *RT* data shown in Supplementary Fig. 1f are measured at 11-ID-C@APS, GPPD@CSNS and ECHIDNA@ANSTO for $MgAg_{0.97}Sb_{0.99}$ sample, respectively.

Supplementary Table 1: Refined parameters of $MgAg_{0.97}Sb_{0.99}$ and $MgAg_{0.965}Ni_{0.005}Sb_{0.99}$ at 20, 50, 100, 200, 300, 350, 400, 450 and 500 K.

T/K	a&b/Å	c/Å	c/a	Mg			Sb			R_{wp}/%	χ^2
$MgAg_{0.97}Sb_{0.99}$:				x	y	z	x	y	z		
20	9.1366(2)	12.6251(3)	1.38182(7)	-0.0268(4)	0.2866(3)	0.1176(4)	0.2384(3)	0.4771(4)	0.1115(3)	1.10	1.93
50	9.1370(2)	12.6309(3)	1.38239(7)	-0.0269(4)	0.2864(3)	0.1175(4)	0.2379(3)	0.4773(4)	0.1116(3)	1.39	2.00
100	9.1383(2)	12.6353(3)	1.38268(7)	-0.0273(4)	0.2863(3)	0.1180(4)	0.2381(3)	0.4774(4)	0.1109(3)	0.71	1.99
200	9.1429(2)	12.6499(3)	1.38358(7)	-0.0278(4)	0.2867(3)	0.1186(3)	0.2387(3)	0.4786(4)	0.1103(3)	1.52	1.82
300	9.1686(2)	12.7057(3)	1.38578(7)	-0.0262(4)	0.2890(3)	0.1068(3)	0.2409(3)	0.4810(4)	0.1189(3)	2.83	1.89
350	9.1774(2)	12.7258(3)	1.38665(7)	-0.0269(4)	0.2887(3)	0.1058(3)	0.2409(3)	0.4827(4)	0.1194(3)	2.65	1.93
400	9.1869(2)	12.7476(3)	1.38758(7)	-0.0268(4)	0.2890(3)	0.1047(3)	0.2418(3)	0.4851(4)	0.1188(3)	1.04	1.76
450	9.1949(2)	12.7689(3)	1.38869(7)	-0.0265(4)	0.2890(3)	0.1038(3)	0.2422(3)	0.4866(4)	0.1191(3)	1.82	1.90
500	9.2006(2)	12.7873(3)	1.38983(7)	-0.0263(4)	0.2890(3)	0.1029(3)	0.2428(3)	0.4883(4)	0.1192(3)	2.89	2.04
$MgAg_{0.965}Ni_{0.005}Sb_{0.99}$:											
20	9.1185(2)	12.6218(3)	1.38420(7)	-0.0268(4)	0.2864(3)	0.1176(4)	0.2385(3)	0.4777(4)	0.1117(3)	0.77	2.65
50	9.1190(2)	12.6250(3)	1.38447(7)	-0.0272(4)	0.2860(3)	0.1173(4)	0.2382(3)	0.4780(4)	0.1117(3)	1.54	2.76
100	9.1203(2)	12.6305(3)	1.38488(7)	-0.0270(4)	0.2861(3)	0.1180(4)	0.2383(3)	0.4781(4)	0.1113(3)	1.29	2.85
200	9.1314(2)	12.6577(3)	1.38617(7)	-0.0276(4)	0.2867(3)	0.1185(3)	0.2394(3)	0.4794(4)	0.1105(3)	1.88	2.74
300	9.1527(2)	12.7017(3)	1.38775(7)	-0.0294(4)	0.2880(3)	0.1198(3)	0.2406(3)	0.4825(4)	0.1090(2)	2.50	2.56
350	9.1621(2)	12.7201(3)	1.38834(7)	-0.0303(3)	0.2880(3)	0.1201(3)	0.2415(3)	0.4842(4)	0.1084(2)	1.49	2.83
400	9.1714(2)	12.7398(3)	1.38908(7)	-0.0308(3)	0.2879(3)	0.1197(3)	0.2422(3)	0.4866(3)	0.1077(2)	3.27	3.00
450	9.1793(2)	12.7578(3)	1.38984(7)	-0.0316(3)	0.2877(3)	0.1201(3)	0.2418(3)	0.4894(4)	0.1074(2)	3.12	3.09
500	9.1852(2)	12.7737(3)	1.39068(7)	-0.0312(3)	0.2884(3)	0.1195(3)	0.2428(3)	0.4901(4)	0.1074(2)	3.77	3.33

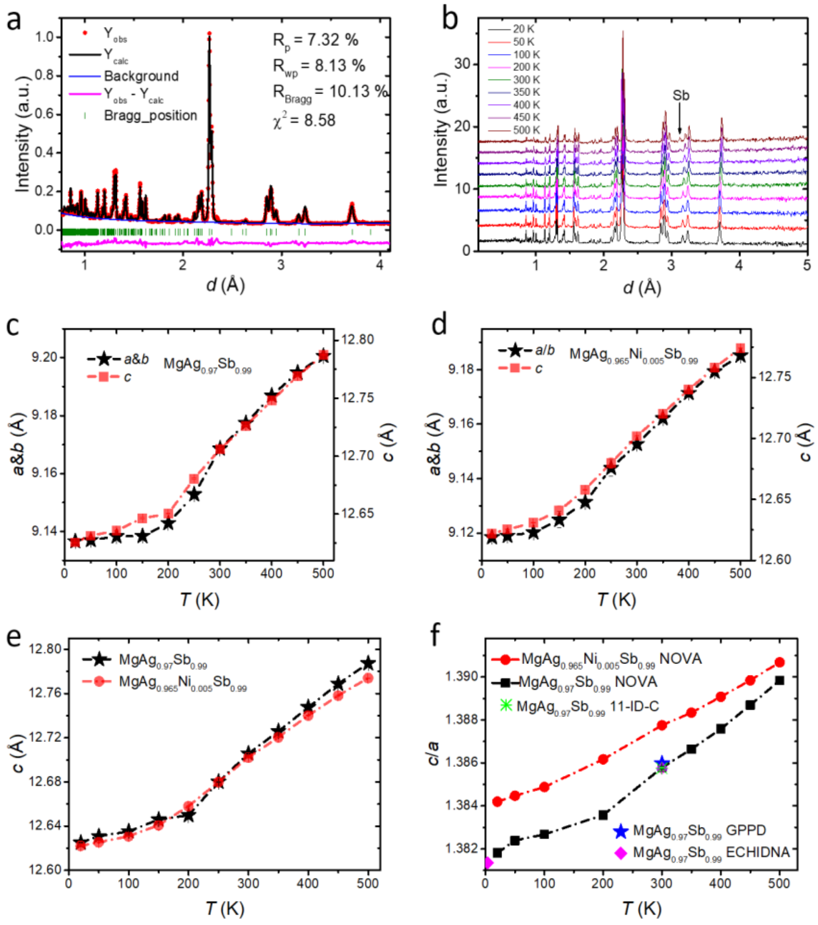

Supplementary Figure 1 | Rietveld refinement results from neutron diffraction data. **a**, Rietveld refinement results of $MgAg_{0.97}Sb_{0.99}$ neutron diffraction data measured at GPPD@CSNS at 250 K. **b**, Temperature-dependent $MgAg_{0.97}Sb_{0.99}$ neutron diffraction data. These data reveal that $MgAg_{0.97}Sb_{0.99}$ maintains the same α-MgAgSb tetragonal structure with a space group of I -4c2 (No.120) over a temperature range from 20 to 500 K. The 450 and 500 K data show the appearance of a small amount of Sb precipitate. Lattice parameters as a function of temperature in **c,** $MgAg_{0.97}Sb_{0.99}$ **d,** $MgAg_{0.965}Ni_{0.005}Sb_{0.99}$. **e,** Comparison of the c lattice parameter between the parent sample $MgAg_{0.97}Sb_{0.99}$ and the Ni-doped sample $MgAg_{0.965}Ni_{0.005}Sb_{0.99}$. **f,** Comparison of the c/a ratio value between $MgAg_{0.97}Sb_{0.99}$ and $MgAg_{0.965}Ni_{0.005}Sb_{0.99}$. From (e) and (f), it can be seen that the Ni-doping shrinks the a lattice parameter while almost no influence on the c lattice parameter. We propose that this anisotropic shrink is related to the smaller Ni^{2+} radius and shorter bond length between Ni and Sb. Error bars are uncertainties from Rietveld refinement and most are smaller than the symbol. a.u., arbitrary units.

Supplementary Table 2: Atomic displacement parameters obtained from Rietveld refinement.

T/K	Uiso_Mg (Å^2)*	Uiso_Ag (Å^2)	Uiso_Sb (Å^2)	R_{wp} (%)	χ^2
$MgAg_{0.97}Sb_{0.99}$:					
20	0.41(6)	0.04(4)	0.29(6)	1.10	1.93
50	0.44(6)	0.09(4)	0.25(6)	1.39	2.00
100	0.47(6)	0.10(4)	0.27(6)	0.71	1.99
200	0.58(7)	0.13(4)	0.45(6)	1.52	1.82
300	0.63(7)	0.62(5)	0.63(6)	2.83	1.89
350	0.85(8)	0.67(6)	0.77(6)	2.65	1.93
400	1.25(9)	0.91(6)	1.07(6)	1.04	1.76
450	1.56(9)	1.03(6)	1.26(6)	1.82	1.90
500	1.60(1)	1.41(6)	1.33(7)	2.89	2.04
$MgAg_{0.965}Ni_{0.005}Sb_{0.99}$:					
20	0.40(6)	0.04(4)	0.29(5)	0.77	2.65
50	0.41(6)	0.09(4)	0.26(5)	1.54	2.76
100	0.45(6)	0.07(4)	0.30(6)	1.29	2.85
200	0.52(7)	0.21(4)	0.41(6)	1.88	2.74
300	0.63(7)	0.47(5)	0.80(6)	2.50	2.56
350	0.79(7)	0.79(5)	1.08(7)	1.49	2.83
400	1.07(7)	0.92(5)	1.29(7)	3.27	3.00
450	1.21(8)	1.15(6)	1.51(7)	3.12	3.09
500	1.62(9)	1.21(6)	1.58(7)	3.77	3.33

* Here, $Uiso = 8\pi^2\mu^2$, where μ^2 is the root mean square deviation of atoms from its equilibrium position in Å^2.

Supplementary Note 2: Static local structure distortion from the pair distribution function (PDF) analysis.

The Mg-Sb band distances d1-d6 (Fig. 2b) in the distorted Mg-Sb rocksalt sublattice which are extracted by the PDF analysis of the neutron total scattering data in $MgAg_{0.965}Ni_{0.005}Sb_{0.99}$ at 300 K, are 2.86(2), 2.92(1), 3.01(2), 3.30(2), 3.438(9), and 3.90(2) Å, respectively. The Mg atoms are very weakly bonded with the Sb atoms, having nearest-neighbor distances between 2.86(2) and 3.90(2) Å, slightly larger than the summation of the individual atomic radii (Mg: 1.60 Å and Sb: 1.44 Å).

Supplementary Note 3: Phonon spectrum from a*b initio* calculation compared with INS data.

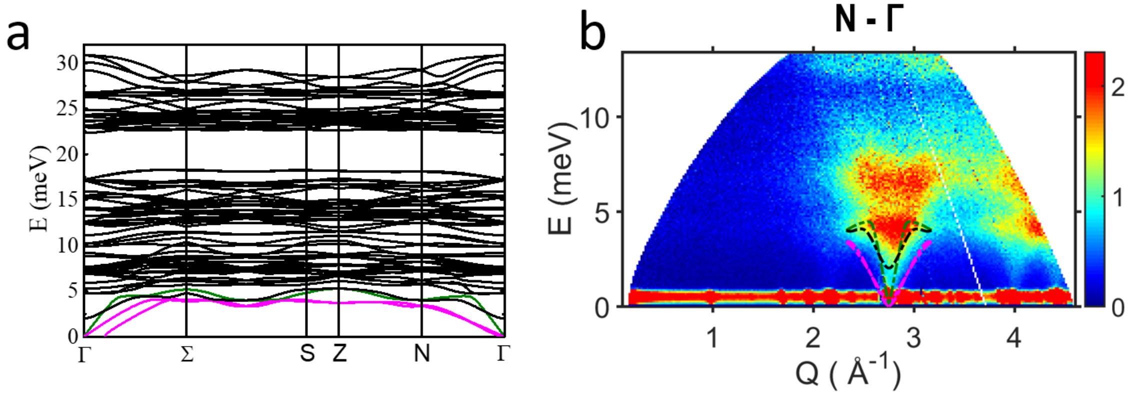

Supplementary Figure 2 | Calculated phonon spectrum of α-MgAgSb in comparison with the pattern measured by INS. a, The phonon spectrum of α-MgAgSb with the k-path is along Γ(0 0 0)-Σ(-0.380594 0.380594 0.380594)-S(0.380594 0.619406 -0.380594)-Z(0.500 0.500 -0.500)-N(0.000 0.500 0.000)-Γ. The overlap of longitudinal acoustic and longitudinal optic modes in MgAgSb could lead to enhanced interaction between the high-frequency acoustic modes and the low-frequency optic modes. **b,** The transverse acoustic, longitudinal acoustic and low-frequency optic phonon branches along the Γ-N direction are superimposed on the INS-mapped $B(\mathbf{Q}, E)$ pattern. The transverse acoustic phonons are mostly suppressed and thus the INS-measured low-energy modes mainly result from longitudinal acoustic phonons.

Supplementary Note 4: Phonon spectrum of the adjusted symmetric structure from a*b initio* calculation.

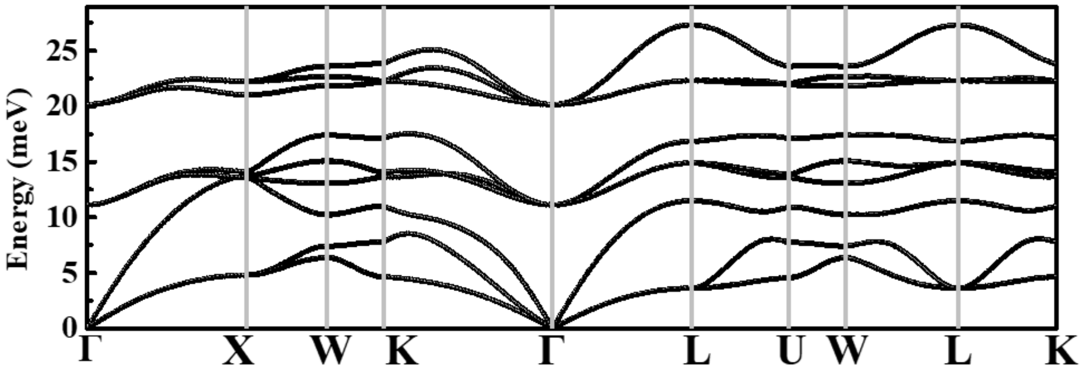

Supplementary Figure 3 | Calculated phonon spectrum of the symmetric structure shown in Fig. 3f. Here, the phonon modes around 5 meV are due to the transverse phonons while the longitudinal acoustic phonon modes reach a high-energy level.

Supplementary Note 5: Magnifications of areas of *B*(Q, *E*).

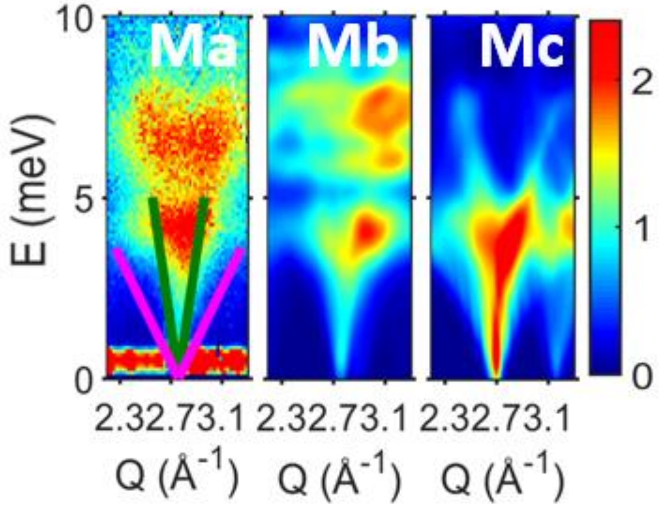

Supplementary Figure 4 | Magnifications of areas of *B*(Q, *E*) at the *Q* from 2.2 to 3.3 Å^{-1} and *E* from 0 to 10 meV region in Fig. 3a (Ma), Fig. 3b (Mb) and Fig. 3c (Mc), show their differences clearly visible.

Supplementary Note 6: Phonon softening from *ab initio* calculations.

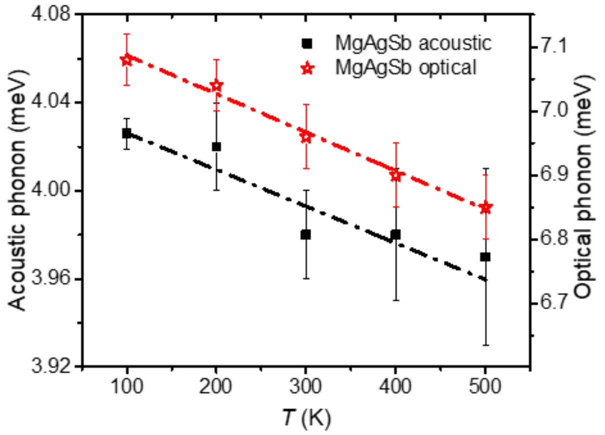

Supplementary Figure 5 | Calculated phonon peak positions from the DOS of the *α*-MgAgSb phase. Temperature dependence of the peak position of the acoustic phonons and low-frequency optical phonons fitted from the *ab initio* results, which are calculated based on the temperature-dependent structure parameters refined from the neutron powder diffraction data. These data show a weaker softening as a function of temperature than that of the fitting from INS data shown in Fig. 4c. The phonon softening ratio values are shown in Supplementary Table 3. Error bars result from the statistical uncertainties in fitting the phonon peaks.

Supplementary Table 3: Phonon softening ratio from the fitting of *ab initio* calculation data.

Phonon softening ratio: the slope of the fitting line	Acoustic peak (meV/K)	Optical peak (meV/K)
$MgAg_{0.97}Sb_{0.99}$ / INS	-2.740*10^{-4}	-1.14*10^{-3}
$MgAg_{0.965}Ni_{0.005}Sb_{0.99}$ / INS	-2.784*10^{-4}	-1.21*10^{-3}
α-MgAgSb / Simulation	-1.665*10^{-4}	-5.999*10^{-4}

Supplementary Note 7: Hall measurements demonstrate the Ni doped in Ag site.

For the confirmation of Ni goes in Ag site. Here, the ~0.5% Ni-doping cannot be determined by the Rietveld refinement limited by the resolution. We further performed Hall measurements and the carrier concentrations (n_H) were determined (Supplementary Table 4). The n_H is increased more than 10% after Ni-doping, which demonstrated that the Ni^{2+} doped in Ag^{1+} site instead of Mg^{2+}. While the resistivity increase is due to the decreasing of carrier mobility.

Supplementary Table 4: Resistivity, carrier mobility and carrier concentrations (n_H) determined from Hall measurements at *RT*.

	Resistivity (Ω*cm)	Mobility (cm²/V/s)	n_H (10^{19}/cm³)
$MgAg_{0.97}Sb_{0.99}$:			
Heat annealing	0.002054	77.6	+3.916
	0.002054	74.4	+4.086
	0.002058	76.2	+3.979
Mean value	0.002056	76.1	+3.994
$MgAg_{0.965}Ni_{0.005}Sb_{0.99}$:			
Heat annealing	0.003082	49.1	+4.121
	0.003083	45.0	+4.498
	0.003080	43.6	+4.652
Mean value	0.003082	45.9	+4.424

Supplementary Note 8: Three-phonon scattering phase space for MgAgSb.

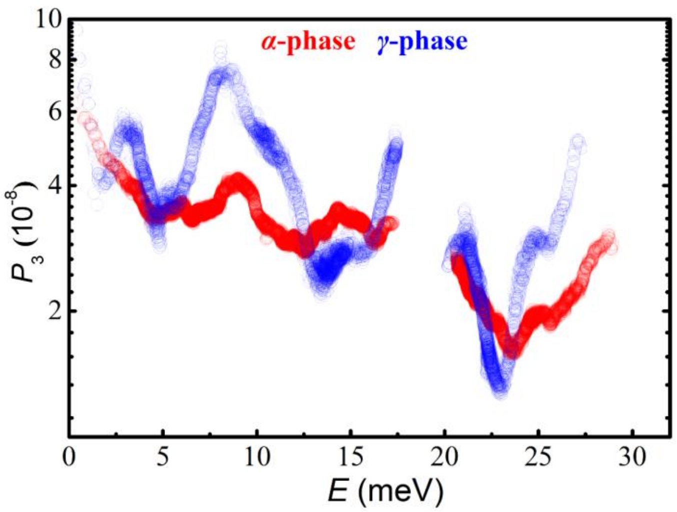

Supplementary Figure 6 | Calculated P_3 of α-MgAgSb and in the high-symmetry structure γ-MgAgSb. The average P_3 of α-MgAgSb is much lower than that of γ-MgAgSb, especially for the transverse acoustic modes and longitudinal acoustic modes in the low-frequency region (<10 meV). This greatly weakens the intrinsic phonon-phonon scattering, increases the phonon lifetime of these phonon modes (Fig. 5c), and gives rise to the decrease of phonon anharmonicity of γ-MgAgSb. However, the static local structure distortion fully scattered the transverse acoustic phonons and accordingly suppress the phonon transport in α-MgAgSb. As a result, the mechanisms of static local structure distortion suppresion transverse phonons in α-MgAgSb and giant anharmonicity in γ-MgAgSb lead to the nearly equal κ_{lat} of the two polymorphisms.

The three-phonon scattering phase space (P_3) is a quantity describing the space available for the three-phonon process allowed by the conservations of the energy and momentum[2,3]. Generally, a higher value of P_3 implies more space available for the three-phonon scattering processes, which typically is an indicator of lower phonon lifetimes. The mode-dependent phase space $P_3(\omega_j(\mathbf{q}))$ is defined by[4]:

$$P_3\left(\omega_j(\mathbf{q})\right) = \frac{1}{\Omega_{BZ}}\left[\frac{2}{3}D_j^{(+)}(\mathbf{q}) + \frac{1}{3}D_j^{(-)}(\mathbf{q})\right],$$

where Ω_{BZ} is the volume of Brillouin zone (BZ); $D_j^{(\pm)}(\mathbf{q})$ are two-phonon densities of states for absorption (+) and emission (−) processes. The P_3 values as a function of

frequency ω_j evaluated over the *BZ* for *α*- and *γ*-MgAgSb at 300 K were shown in Supplementary Fig. 6. It is clear that in the low-frequency region (<10 meV) the P_3 values of *α*-MgAgSb are much lower than those of *γ*-MgAgSb, indicating that easier three-phonon scattering processes occur in *γ*-MgAgSb. This explains why *α*-MgAgSb has a much longer relaxation time than *γ*-MgAgSb (Fig. 5c).

Supplementary Note 9: Lattice thermal conductivity of the high-symmetry structure *γ*-MgAgSb.

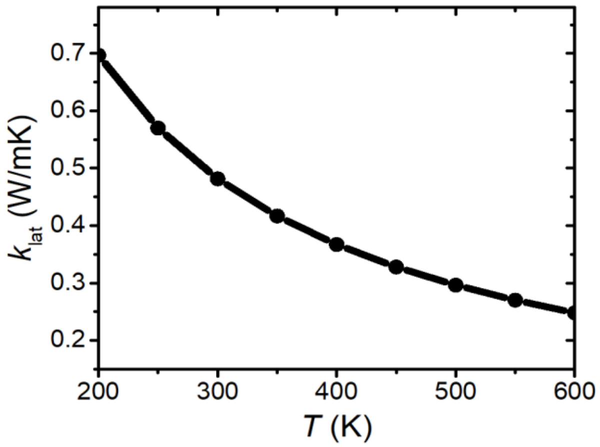

Supplementary Figure 7 | Calculated temperature-dependent κ_{lat} of the *γ*-MgAgSb.

Supplementary Note 10: Partial phonon DOS contributed from different atoms.

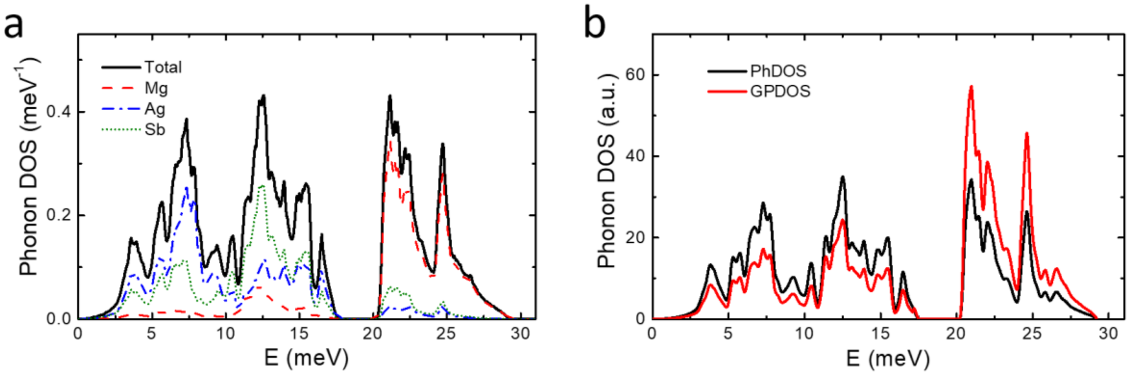

Supplementary Figure 8 | Calculated phonon DOS of the *α*-MgAgSb phase. a, The partial phonon DOS contributed from different atoms are separated. **b,** For comparison with INS experiment data, the neutron scattering cross section weighting factor is added in the GPDOS data. a.u., arbitrary units.

Supplementary References

1. Chen, J. *et al.* The general purpose powder diffractometer at CSNS. *Phys. B Condens. Matter* **551**, 370–372 (2018).

2. Lindsay, L. & Broido, D. A. Three-phonon phase space and lattice thermal conductivity in semiconductors. *J. Phys. Condens. Matter* **20**, 165209 (2008).

3. Lee, S. *et al.* Resonant bonding leads to low lattice thermal conductivity. *Nat. Commun.* **5**, 3525 (2014).

4. Li, W., Carrete, J., A. Katcho, N. & Mingo, N. ShengBTE: A solver of the Boltzmann transport equation for phonons. *Comput. Phys. Commun.* **185**, 1747–1758 (2014).
